# Reduction of ferulic acid as an electron acceptor under anaerobic conditions by the heterofermentative lactic acid bacterium *Weissella cibaria*

**DOI:** 10.1128/aem.00111-26

**Published:** 2026-03-30

**Authors:** Ryoji Mitsui, Hikaru Maruko, Riyo Awa, Fusako Kawamoto, Daigo Iwasaki, Yosuke Nishitani, Hiroshige Kuwahara, Takanori Yano

**Affiliations:** 1 Department of Bioscience, Faculty of Life Science, Okayama University of Science, Ridai-cho, Kita-ku 13019, Okayama, Japan; 2 Research Center, Maruzen Pharmaceuticals Co., Ltd. 201180 , Fukuyama, Hiroshima, Japan; Universita degli Studi di Napoli Federico II, Portici, Italy

**Keywords:** *Weissella cibaria*, ferulic acid, HMPA, FarA, FarB, redox balance, lactic acid bacteria, heterolactic fermentation

## Abstract

When the heterofermentative lactic acid bacterium *Weissella cibaria* JCM12495 was cultivated anaerobically in medium supplemented with 3-hydroxy-4-methoxycinnamic acid (ferulic acid), all the added ferulic acid was converted to 3-(4-hydroxy-3-methoxyphenyl)propionic acid (HMPA, dihydroferulic acid) via reduction of the propenoic acid side chain. Complete conversion to HMPA was achieved within 16 h, even at an initial ferulic acid concentration of 10 mM. Concurrently, the production of typical heterolactic fermentation products, such as lactic acid and ethanol, was suppressed, while acetic acid formation was increased. Transcriptomic analysis revealed that only two genes were significantly induced by ferulic acid addition, and these genes form an operon in the genome. We individually expressed these two genes in *Escherichia coli* and assessed their enzymatic activities *in vitro* against ferulic acid by identifying reaction products using liquid chromatography quadrupole time-of-flight mass spectrometry. When ferulic acid and acetyl-CoA were added to a reaction mixture containing the first expressed protein, feruloyl-CoA was detected as the product. Subsequently, when the generated feruloyl-CoA was incubated with the second expressed protein in the presence of FAD and NADH, the formation of HMPA was confirmed. These results demonstrate that the two genes encode a ferulate CoA-transferase (FarA), which catalyzes the CoA ligation of ferulic acid, and a feruloyl-CoA reductase (FarB), which reduces the propenoic acid side chain of feruloyl-CoA to produce HMPA. Our findings reveal an anaerobic respiratory pathway coupled to heterolactic fermentation, in which ferulic acid acts as an electron acceptor, thereby influencing carbohydrate utilization during heterolactic fermentation.

**IMPORTANCE:**

Probiotics and lactic acid bacteria (LAB) in fermented foods are of significant interest due to their health-promoting effects. This study shows that *Weissella cibaria*, a common LAB found in plant-derived fermented foods, uses ferulic acid, a compound bound to dietary fibers such as arabinoxylans, as an external electron acceptor for anaerobic redox balancing. This metabolic strategy is associated with redox regulation during heterolactic fermentation and leads to the production of 3-(4-hydroxy-3-methoxyphenyl)propionic acid, a metabolite that has been reported to have health benefits in humans. These findings reveal an unrecognized link between LAB metabolism, dietary polyphenol conversion, and potential probiotic functions, providing new insight into how gut-associated microbes contribute to host health.

## INTRODUCTION

Lactic acid bacteria (LAB) are a group of Gram-positive microorganisms that anaerobically convert sugars into lactic acid with high yield. They are broadly classified into two groups based on their fermentation pathways: homofermentative lactic acid fermentation, in which pyruvate serves as the primary electron acceptor to produce lactic acid; and heterofermentative lactic acid fermentation, in which lactic acid is produced from pyruvate, while ethanol is additionally generated through the reduction of acetaldehyde derived from acetyl-CoA, serving as an alternative electron acceptor.

In both homo- and heterofermentative pathways, the physiological role of lactic acid production in LAB is to maintain intracellular redox balance by regenerating NAD^+^ under anaerobic growth conditions. This is achieved by using pyruvate as the principal electron acceptor to reoxidize NADH generated during glycolysis (1). In heterofermentative lactic acid fermentation, acetaldehyde derived from acetyl-CoA also acts as an electron acceptor, leading to the formation of ethanol.

Several recent reports describe mechanisms by which lactic acid bacteria regulate redox balance through the utilization of externally supplied compounds as alternative electron acceptors. For instance, the homofermentative lactic acid bacterium *Lactiplantibacillus plantarum* (formerly *Lactobacillus plantarum*) is known to utilize NADH-dependent enzymes HcrA and HcrB to reduce the propenoic acid side chains of hydroxycinnamic acids (e.g., *p*-coumaric acid, ferulic acid, caffeic acid, and sinapinic acid), which are abundant in fruits, vegetables, and cereals. The resulting propionic acid derivatives are subsequently secreted extracellularly. These reduction pathways are particularly significant because they allow redox balance to be maintained independent of pyruvate as the terminal electron acceptor in carbohydrate metabolism. This process enhances the efficient utilization of carbon sources and confers a growth advantage under anaerobic conditions. Additionally, the activity of phenolic acid decarboxylase (PDC) has been reported in certain strains, which results in the formation of corresponding vinyl derivatives (2–5). Similarly, in heterofermentative lactic acid bacteria, such as *Lactobacillus curvatus* and *Weissella cibaria*, the reduction of hydroxycinnamic acids as external electron acceptors has been demonstrated to significantly increase the intracellular ATP yield per glucose consumed (3). However, since homologs of *hcrA* and *hcrB* have not been identified in these heterofermentative species, this suggests the involvement of novel reductases in this pathway (6). Based on these findings, it is presumed that in the human intestinal tract, dietary hydroxycinnamic acids are reduced by anaerobic bacteria, including LAB, and that these reduction reactions contribute to the bacteria’s adaptive growth under anaerobic conditions. Chlorogenic acid, for example, was first discovered by Payen in 1864 in green coffee beans. It is a naturally occurring polyphenol widely distributed in fruits and vegetables. Chlorogenic acid is an ester composed of caffeic acid and quinic acid, and it exhibits a broad range of physiological activities in humans (7, 8). Upon consumption of coffee, approximately one-third of chlorogenic acid is absorbed in the small intestine, while the remaining two-thirds reach the large intestine (9). Chlorogenic acid absorbed in the small intestine is excreted in urine either intact or as a sulfate conjugate. In contrast, the fraction that reaches the large intestine interacts with the gut microbiota, leading to the formation of microbial metabolites detected in human urine. These metabolites include dihydrocaffeic acid and its sulfate, as well as 3-(4-hydroxy-3-methoxyphenyl) propionic acid (HMPA, dihydroferulic acid) and its sulfate (10). These observations suggest the presence of both microbial esterases, which hydrolyze chlorogenic acid, and reductases in the colonic microbiota, which convert the resulting caffeic acid and ferulic acid into their respective dihydro derivatives (11). Many polyphenols are known to exhibit beneficial physiological activities in humans. It has been reported that various polyphenols are metabolized by the gut microbiota to produce phenolic acids, including dihydrocinnamic acid derivatives (12). For example, *in vitro* colonic fermentation models inoculated with human fecal microbiota have shown that HMPA is a major metabolite of curcumin (13). Similarly, HMPA formation and excretion have been observed following the intake of hesperetin-7-O-rutinoside from orange juice (14), and HMPA has also been detected among the urinary metabolites of γ-oryzanol in rats (15). These findings suggest that the dihydrocinnamic acid derivatives generated from dietary polyphenols by microbial metabolism in the gut may be the actual bioactive agents responsible for their physiological effects in humans and animals.

The G-protein coupled receptor 41 (GPR41) (also known as FFAR3), which is highly expressed in the intestines of humans and other mammals, is known as a receptor for short-chain fatty acids and plays a key role in the regulation of energy balance and metabolism (16). In mouse studies, HMPA has been reported to exhibit affinity for GPR41 and to stimulate fatty acid catabolic pathways. Comparative analyses also showed that HMPA possesses a higher affinity for GPR41 than its precursor, ferulic acid, and that activation of this receptor by HMPA exerted anti-obesity effects and improved hepatic lipid metabolism in mice fed a high-fat diet (17). Taken together, these findings suggest that HMPA, as a key microbial metabolite formed through the interaction between the gut microbiota and dietary polyphenols, may have significant implications for host health.

In this study, we identified a high level of HMPA-producing activity from ferulic acid in *Weissella cibaria*, the type strain isolated from the Malaysian fermented food *chili bo* (18). We successfully identified two previously uncharacterized enzymes responsible for this reductive activity: ferulate CoA-transferase and feruloyl-CoA reductase. *W. cibaria* is a heterofermentative lactic acid bacterium frequently isolated from fermented foods and is known for its probiotic properties. These findings suggest that the intake of this bacterium may contribute to HMPA production in the human gut. Therefore, the primary aim of the present study was to fully characterize the novel feruloyl-CoA reductase pathway in *W. cibaria*, investigate its contribution to HMPA production, and discuss the implications of this probiotic-mediated metabolism for host health.

## RESULTS

### Reductive conversion of ferulic acid to HMPA in *Weissella cibaria*

**Fig 1:**
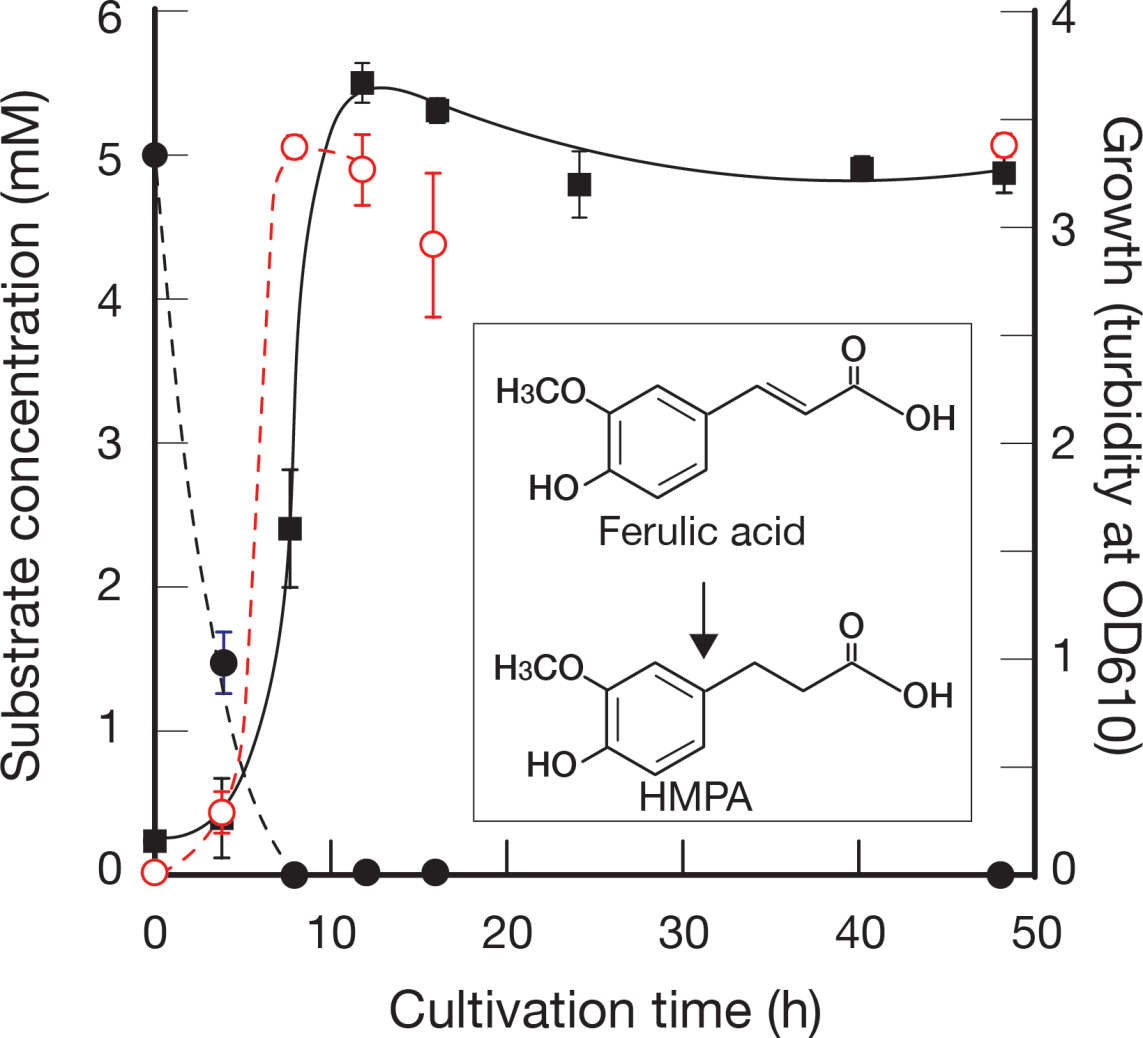
Conversion of ferulic acid to HMPA by *Weissella cibaria*. The filled circles (●) represent ferulic acid, and the open circles (○) represent HMPA. The filled squares (■) indicate the optical density at 610 nm (OD₆₁₀) of *W. cibaria* cultures. Error bars represent standard deviations (*n* = 3).

*Weissella cibaria* JCM12495^T^ is the type strain of *W. cibaria*, originally isolated by Björkroth et al., and is a heterofermentative lactic acid bacterium (18). When cultured statically at 37℃ in MRS medium supplemented with 5 mM ferulic acid, the strain reached peak growth at approximately 12 h (Fig. 1). The added ferulic acid was gradually converted to HMPA during growth and was no longer detectable after approximately 8 h. This result indicates that *W. cibaria* possesses the ability to reduce the double bond in the propenoic side chain of ferulic acid under anaerobic conditions. The resulting HMPA remained in the culture supernatant even after extended incubation and was not further metabolized. Notably, no production of 4-vinylguaiacol (4-VG), a known decarboxylation product observed in *Lactiplantibacillus plantarum* (formerly *Lactobacillus plantarum*), was detected (2, 19). The molar yield of HMPA was nearly 100%, indicating that, like the *W. cibaria* strain reported by Filannino et al. (6), this strain utilizes ferulic acid as an external electron acceptor for redox balance during anaerobic growth.

### Identification of ferulic acid-inducible genes

**Fig 2:**
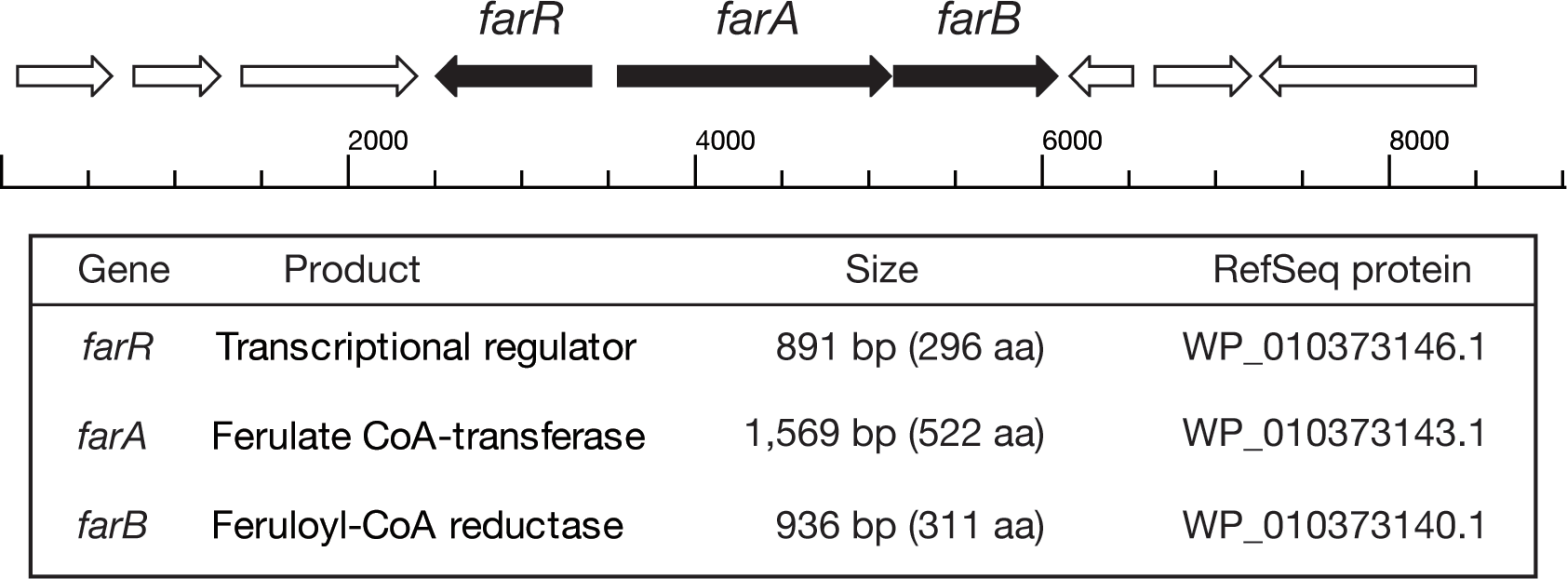
Ferulic acid-inducible operon involved in ferulic acid reduction in *Weissella cibaria*. The operon consists of two genes, *farA* and *farB*, encoding a ferulate CoA-transferase and a feruloyl-CoA reductase, respectively. Arrows indicate the direction of transcription. The genes are located on contig 3 (NZ_BJEF01000003) of the *W. cibaria* JCM12495 genome (GenBank assembly GCF_005405525.1).

Genome analysis of the *Weissella cibaria* type strain JCM12495 (GCF_005405525.1) revealed an approximately 2.3 Mb genome sequence. No homologs of known ferulic acid reductase, genes such as *hcrA* or *hcrB*, were identified in the annotated genome. This suggested the presence of a novel reductase involved in HMPA production. To identify such candidate genes, we focused on those whose expression was induced in response to ferulic acid. Total RNA was extracted from cells cultured in the presence and absence of ferulic acid, and transcriptome analysis was performed. Comparison of gene expression levels revealed that only a limited number of genes exhibited significant changes. Among them, two genes showed the most pronounced response, with their TPM values increasing approximately 50-fold upon the addition of ferulic acid (Table S1). These two genes were predicted to be organized in an operon (Fig. 2). Two novel ferulic acid-reducing enzyme genes were predicted based on genome analysis and subsequently identified. The upstream gene, designated *farA* (RefSeq protein accession no. WP_010373143.1), is 1,569 bp in length and belongs to the acyl-CoA:acetate/3-ketoacid CoA transferase family. It shows sequence similarity to YdiF from *Escherichia coli* (20). Since YdiF is known to act on short-chain acyl-CoAs, FarA was predicted to catalyze the attachment of CoA to the propenoic side chain of ferulic acid. The downstream gene, named *farB* (RefSeq protein accession no. WP_010373140.1), is 936 bp in length and shows similarity to enoyl-ACP reductases involved in fatty acid biosynthesis. Based on this, FarB is expected to reduce the CoA-conjugated propenoic side chain, resulting in formation of a saturated side chain structure.

### Detection of ferulic acid reduction activity and identification of the product

**Fig 3:**
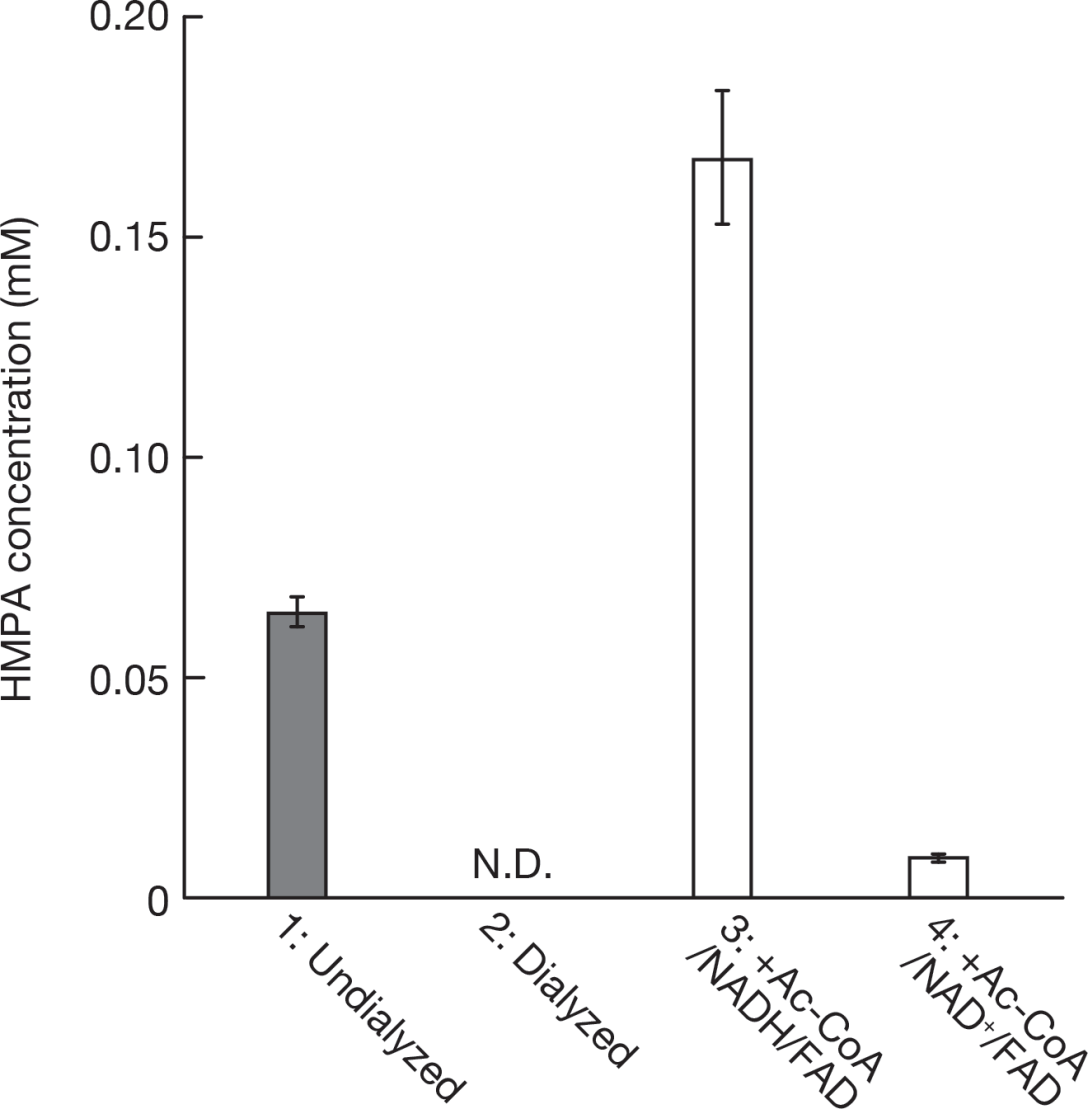
Factors required for ferulic acid reduction activity. Gray bars represent reactions using undialyzed crude enzyme extracts (Condition 1), and white bars represent reactions using dialyzed enzyme extracts (Conditions 2–4). Conditions 1 and 2: Ferulic acid (2 mM). Condition 3: Ferulic acid (2 mM) was incubated with acetyl-CoA, NADH, and FAD (each 2 mM). Condition 4: Same as Condition 3, except that NADH was replaced with NAD^+^. N.D., HMPA not detected. Reaction mixtures were incubated for 2 h at 37°C. Error bars represent standard deviations (*n* = 3).

Crude enzyme extracts prepared from *W. cibaria* cells cultured in the presence of ferulic acid were used to examine ferulic acid reductase activity and to identify the resulting product (Fig. 3). Based on preliminary experiments, the reaction was performed at pH 6.5 and 35°C, which were close to the optimal conditions for FarA activity (Fig. S1). Accordingly, a reaction mixture containing 2 mM ferulic acid and the crude enzyme extract of *W. cibaria* was prepared in 50 mM potassium phosphate buffer (pH 6.5). After incubation at 35℃ for 2 h, the reaction mixture was extracted with ethyl acetate and analyzed by HPLC, resulting in the detection of HMPA as the reduction product. In contrast, when the crude extract was dialyzed to remove small compounds, HMPA was no longer detected. This result suggests that other substrates or cofactors, such as small-molecule compounds, may be involved in the reduction of ferulic acid. Therefore, we added NAD^+^, NADH, and FAD, cofactors predicted to be involved in redox regulation during heterolactic fermentation, to the reaction mixture containing acetyl-CoA and the dialyzed crude enzyme extract. Although none of the cofactors induced HMPA production when added individually, a reaction mixture containing the dialyzed crude enzyme extract supplemented with 2 mM each of NADH, FAD, and acetyl-CoA yielded a higher concentration of HMPA than that obtained using the non-dialyzed crude extract. In contrast, when NAD^+^ was used instead of NADH, HMPA production was barely detectable. These results suggest that ferulic acid may be utilized in redox balance regulation, potentially acting competitively with heterolactic fermentation by serving as an electron acceptor to regenerate NAD^+^ required for glycolysis.

### Induction of ferulate CoA-transferase by hydroxycinnamic acids

**Fig 4:**
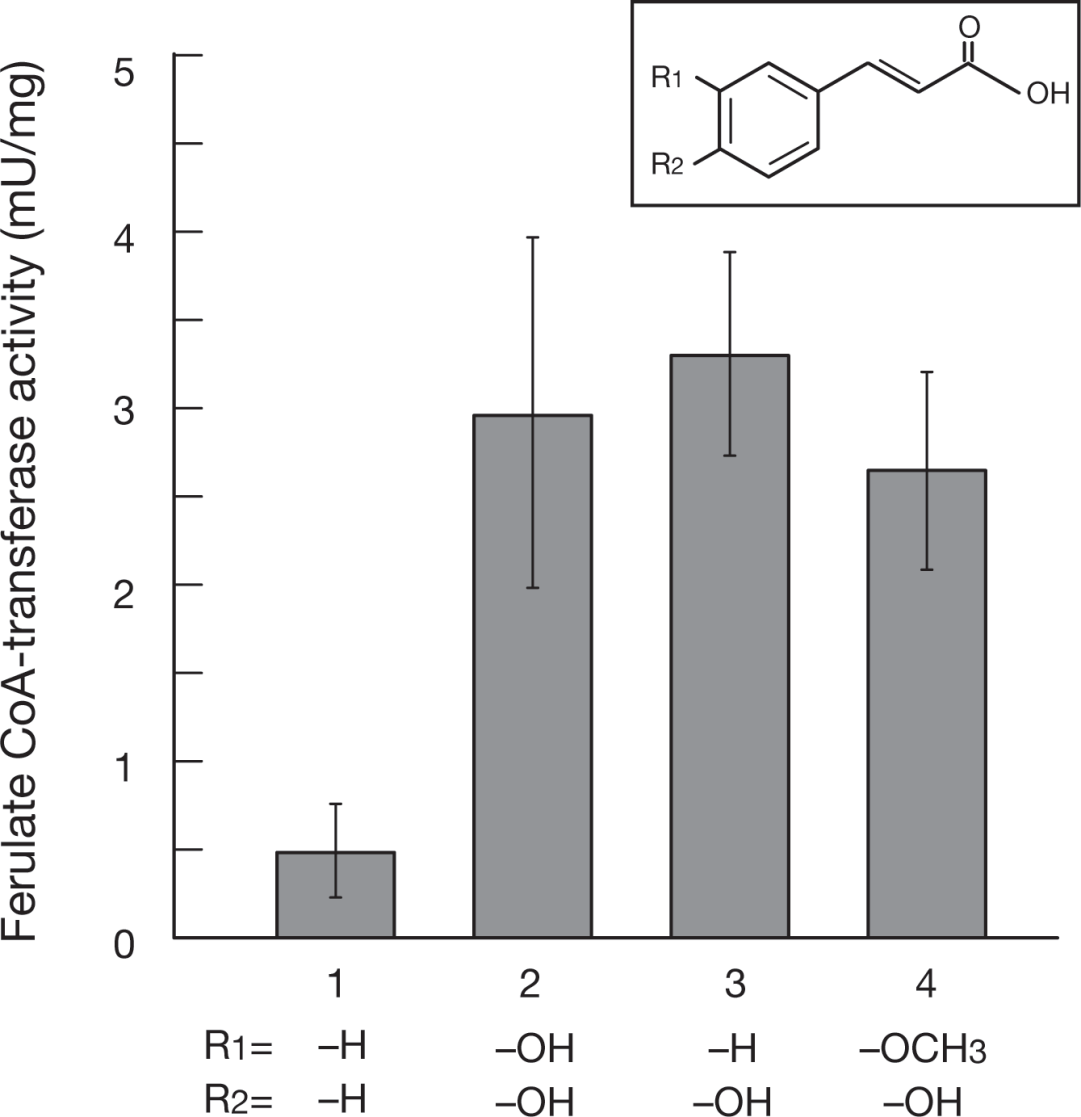
Induction of FarA activity by cinnamic acid derivatives. *Weissella cibaria* was cultured for 16 h in MRS medium supplemented with 30 mM of each hydroxycinnamic acid derivative. Crude enzyme extracts were prepared and used to measure FarA activity. R₁ and R₂ indicate the substituents on the aromatic ring of each compound, as shown in the inset. Compounds: 1, cinnamic acid; 2, caffeic acid; 3, *p*-coumaric acid; 4, ferulic acid. Error bars represent standard deviations (*n* = 3).

Ferulate CoA-transferase activity, associated with the induced expression of *farA*, was confirmed by measuring changes in absorbance corresponding to the production of feruloyl-CoA using crude extracts prepared from *W. cibaria* cells cultured in MRS medium supplemented with various hydroxycinnamic acids (Fig. 4). In the enzyme assay, ferulic acid was used as the substrate regardless of the hydroxycinnamic acid used for induction. As a result, ferulic acid, caffeic acid, and *p*-coumaric acid all induced *farA* to a similar extent, whereas cinnamic acid showed only weak induction. These findings suggest that the phenolic hydroxyl group at the para position is involved in the induction of *farA* expression.

### Preparation and identification of ferulic acid-reducing enzymes and their products

To investigate the involvement of two genes in ferulic acid reduction activity, *farA* and *farB* were each expressed under the control of a T7 promoter in *E. coli* as His-tagged fusion proteins. Expression of the target proteins was confirmed by Western blot analysis using an anti-His-tag antibody, which revealed bands corresponding to the molecular weights predicted from the respective gene sequences (Fig. S2). The crude extracts containing the expressed proteins were partially purified by Ni-affinity chromatography and subsequently used for enzymatic analyses.

**Fig 5:**
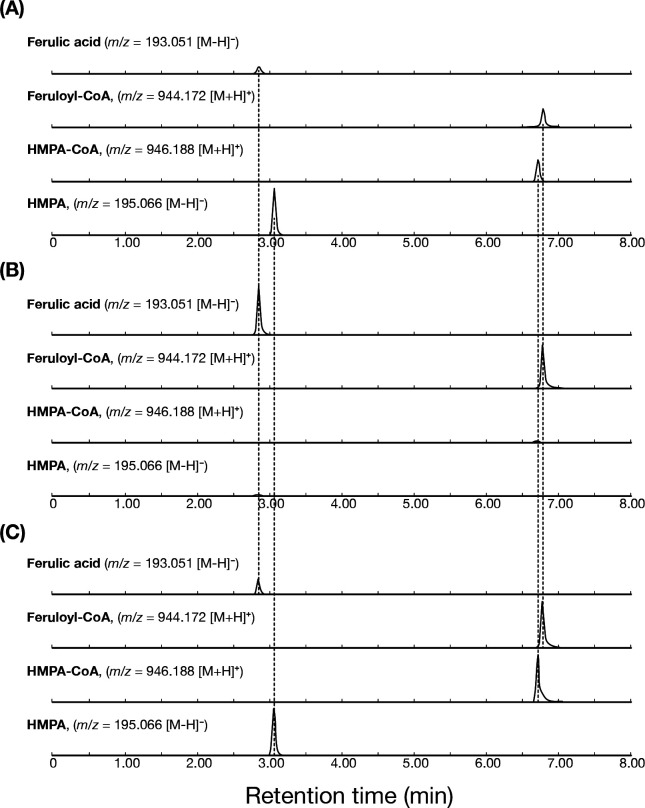
LC-QTOF-MS analysis of metabolites formed from ferulic acid by FarA and FarB. Extracted ion chromatograms were monitored at *m*/*z* values corresponding to ferulic acid, feruloyl-CoA, HMPA-CoA, and HMPA. (**A**) Reaction mixture containing both FarA and FarB. (**B**) Reaction mixture containing FarA alone. (**C**) Reaction mixture with FarA followed by ultrafiltration to remove the enzyme, with subsequent addition of FarB to the filtrate. CoA esters were detected as [M+H]^+^ ions, whereas ferulic acid and HMPA were detected as [M-H] ^-^ions. Dashed vertical lines indicate the retention times of each compound.

**Fig 6:**
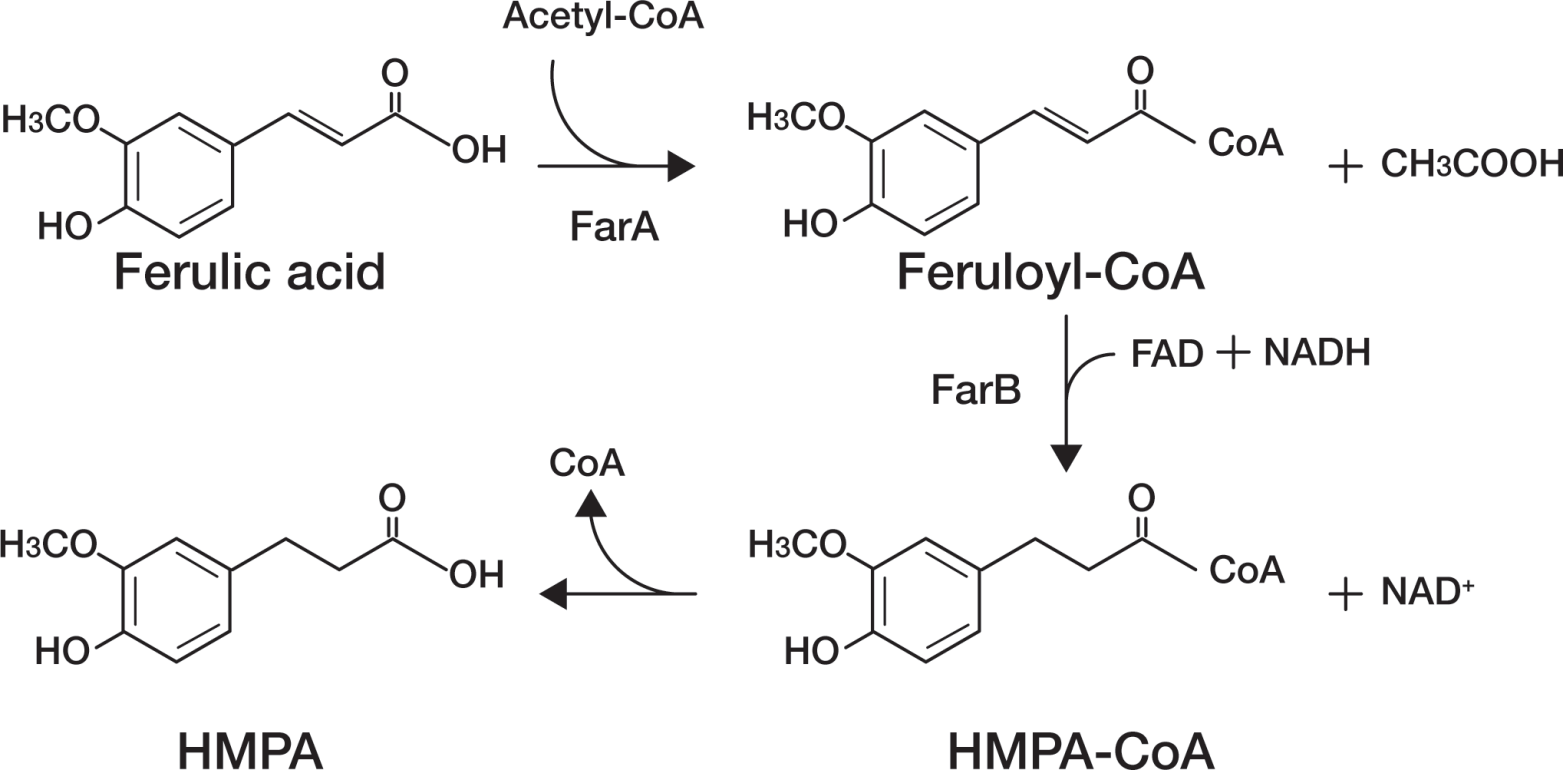
Proposed metabolic pathway for the reduction of ferulic acid to HMPA in *W. cibaria*. Ferulic acid is first converted to feruloyl-CoA by FarA (ferulate CoA-transferase) using acetyl-CoA as the CoA donor. Subsequently, feruloyl-CoA is reduced to HMPA-CoA by FarB (feruloyl-CoA reductase) in the presence of FAD and NADH. Finally, HMPA is formed following the release of CoA from HMPA-CoA.

Reaction products were analyzed qualitatively by liquid chromatography quadrupole time-of-flight mass spectrometry (LC-QTOF-MS), and peaks were assigned based on their *m*/*z* values for identification purposes. In addition to residual ferulic acid and HMPA, peaks in the extracted-ion chromatograms (EICs) corresponding to feruloyl-CoA and HMPA-CoA were detected, with accurate masses matching the calculated exact masses (Fig. 5). A reaction mixture containing ferulic acid, purified FarA and FarB enzymes, acetyl-CoA, NADH, and FAD was incubated for 24 h (Fig. 5A). Under these conditions, the formation of HMPA was observed together with the accumulation of feruloyl-CoA and HMPA-CoA. When the reaction was performed using only purified FarA in the presence of acetyl-CoA, feruloyl-CoA was detected, whereas neither HMPA-CoA nor HMPA was observed (Fig. 5B). To further confirm the sequential reaction, FarA was removed from the reaction mixture after 6 h by ultrafiltration using a 10,000 NMWL cutoff membrane, and purified FarB was subsequently added together with NADH and FAD (Fig. 5C). LC-QTOF-MS analysis provided clear evidence for the presence of both HMPA-CoA and HMPA, indicating that feruloyl-CoA generated by FarA was reduced by FarB to form HMPA-CoA. HMPA was detected as the final product of the reaction, suggesting that HMPA-CoA is subsequently converted to HMPA with the release of CoA. Because no additional enzymes were present in the reaction mixture, the observed formation of HMPA suggests that HMPA-CoA may undergo conversion to HMPA under the assay conditions. However, the mechanism of CoA release from HMPA-CoA was not directly examined in the present study (Fig. 6).

### Effect of ferulic acid addition on fermentation product formation

**Fig 7:**
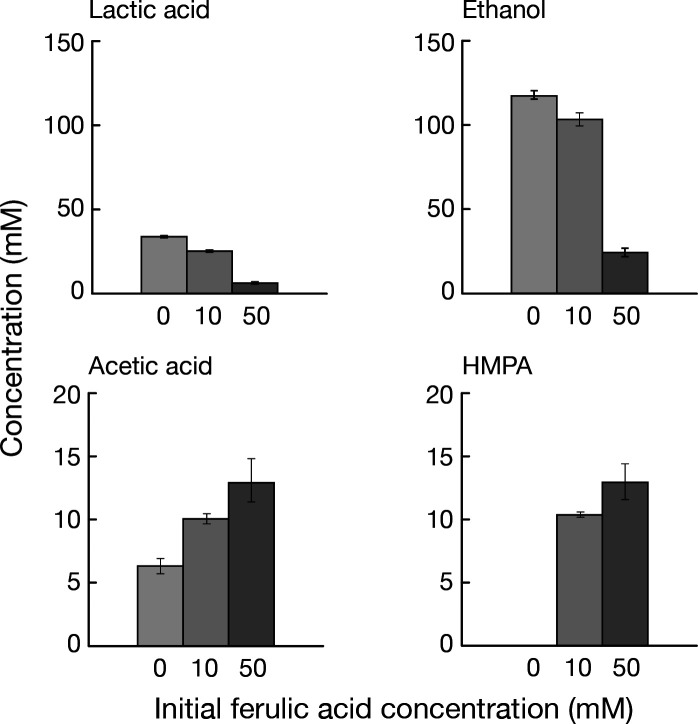
Effect of ferulic acid addition on the production of heterolactic fermentation metabolites in *Weissella cibaria*. *W. cibaria* was cultured anaerobically in MRS medium supplemented with 0, 10, or 50 mM ferulic acid for 16 h. Concentrations of fermentation products (lactic acid, ethanol, and acetic acid) and the ferulic acid reduction product (HMPA) were determined. The results show a decrease in lactic acid and ethanol production as well as an increase in acetic acid and HMPA formation, as the initial ferulic acid concentration increases. Error bars represent standard deviations (*n* = 3).

It was hypothesized that, in the presence of ferulic acid, *W. cibaria* would preferentially reduce ferulic acid over producing lactate and ethanol, thereby oxidizing NADH, contributing to the intracellular redox balance, and enabling more efficient carbon utilization. To examine the effects of ferulic acid on heterolactic fermentation, *W. cibaria* was cultured anaerobically for 16 h in MRS medium supplemented with various concentrations of ferulic acid. After cultivation, the concentrations of lactic acid, acetic acid, and ethanol, typical products of heterolactic fermentation, were quantified, as was HMPA, the reduction product of ferulic acid. To verify that ferulic acid did not inhibit cell growth under these conditions, growth was monitored at different ferulic acid concentrations. No growth inhibition was observed under the tested conditions (Fig. S3). In the absence of ferulic acid, *W. cibaria* produced ethanol as the major heterolactic fermentation product, followed by lactic acid, indicating that ethanol formation was prioritized over lactate production. When cultured in MRS medium with 10 mM ferulic acid, all of the added ferulic acid was converted to HMPA after 16 h of cultivation. Under this condition, the production of lactic acid and ethanol was clearly reduced, whereas acetate production increased. This shift is likely attributable to the CoA transfer reaction between ferulic acid and acetyl-CoA catalyzed by FarA. When the cultivation was performed with an initial concentration of 50 mM ferulic acid, under conditions where ferulic acid was not completely reduced, the production of lactic acid and ethanol was markedly suppressed (Fig. 7). These results suggest that, in the presence of ferulic acid, *W. cibaria* can utilize ferulic acid as an alternative electron acceptor contributing to intracellular redox balance. Once all the ferulic acid is completely reduced to HMPA, the cells appear to return to the normal heterolactic fermentation pathway.

## DISCUSSION

**Fig 8:**
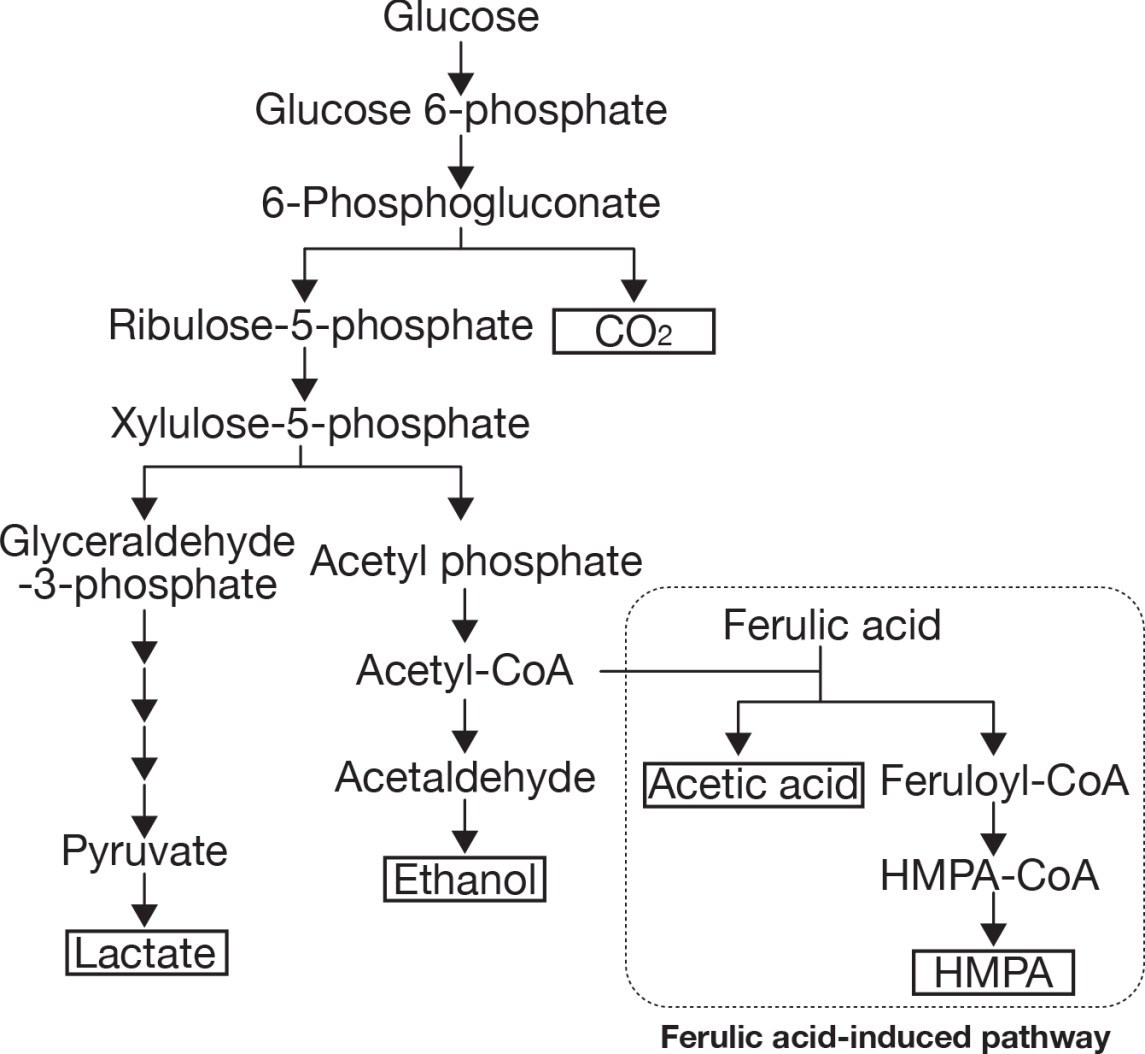
Proposed metabolic model of the ferulic acid reduction pathway coordinated with heterolactic fermentation in *Weissella cibaria*. Compounds enclosed in boxes represent metabolic end products. The dashed area indicates the metabolic route induced by the addition of ferulic acid. Ferulic acid is proposed to function as an alternative electron sink, facilitating NAD^+^ regeneration through the FarB-dependent reduction pathway. This metabolic shift is proposed to divert the flux of acetyl-CoA from ethanol production toward acetic acid formation, potentially enhancing energy yields through increased ATP production.

In *Weissella cibaria*, we identified two genes that are co-transcribed in a polycistronic unit and induced by ferulic acid and related hydroxycinnamic acids. The present study identifies a previously unrecognized ferulic acid reduction pathway in *Weissella cibaria* mediated by two enzymes, FarA and FarB. First, a CoA transferase reaction attaches CoA to the propenoic side chain of ferulic acid, generating feruloyl-CoA. This is followed by a reductase reaction that reduces the double bond to form the propionic acid derivative HMPA-CoA. HMPA was detected as the final product in the reaction mixture, although the mechanism by which CoA is released from HMPA-CoA has not yet been clarified. The addition of ferulic acid altered the production profile of heterolactic fermentation products. Specifically, lactate and ethanol levels were reduced, whereas acetate production was increased. Based on these observations, a proposed pathway for HMPA biosynthesis from ferulic acid is presented in Fig. 8. This pathway appears to compete with the native heterolactic fermentation pathway. Transcriptomic analysis further revealed that ferulic acid supplementation predominantly induced only the two enzymes involved in the HMPA pathway, with little to no upregulation of other fermentative enzymes, suggesting a highly specific transcriptional response. The presence of a reductive metabolic pathway converting ferulic acid to HMPA may serve a critical physiological role in *W. cibaria*. In typical anaerobic fermentation, cells regenerate NAD^+^ by reducing endogenous intermediates, such as pyruvate, leading to the excretion of lactate and ethanol as end products. These products are derived from carbohydrate substrates. In contrast, externally supplied ferulic acid can act as an alternative electron acceptor for redox balancing. By utilizing ferulic acid in this manner, the cell may reduce the production of heterolactic fermentation byproducts derived from sugars and instead channel more carbon flux toward energy-yielding or biosynthetic pathways. This suggests a potential metabolic advantage in environments where ferulic acid or other phenolic compounds are available. A previously reported example of redox balancing using hydroxycinnamic acids is the HcrA/HcrB pathway, which competes with homolactic fermentation in certain *Lactobacillus* strains. Previous genomic analyses of heterofermentative lactic acid bacteria suggested that strains capable of reducing hydroxycinnamic acids do not always harbor known phenolic acid reductases such as HcrB and its homologs. Notably, genes encoding these reductases were not identified in *Weissella cibaria* despite its ability to reduce hydroxycinnamic acids, suggesting the existence of an alternative enzymatic system responsible for this metabolic activity (21). The present study provides a mechanistic explanation for this previously unresolved observation by identifying a distinct reductive pathway mediated by FarA and FarB. Unlike the previously described Hcr system, which directly reduces hydroxycinnamic acids via a flavin-dependent reductase, the Far pathway proceeds through a CoA-thioester intermediate generated by FarA, followed by reduction of the double bond by FarB. This mechanism represents a previously unrecognized metabolic strategy for hydroxycinnamic acid reduction in lactic acid bacteria. Consistent with this mechanistic distinction, sequence comparison and structural prediction analyses revealed no detectable similarity between FarA/B and the Hcr proteins, supporting the notion that these enzymes belong to a distinct functional class. Similar to HcrB, FarB appears to utilize flavin cofactors, such as FAD or FMN, for the oxidation of NADH. Crude extracts from cultures supplemented with ferulic acid exhibited a vivid yellow coloration, indicative of the presence of oxidized flavins (FAD/FMN). This color change was not observed in cultures grown without ferulic acid. These observations indicate that *W. cibaria* actively uses ferulic acid as an external electron acceptor, thereby facilitating more efficient intracellular redox balance.

*W. cibaria* is known as a probiotic lactic acid bacterium frequently isolated from plant-based fermented foods (22, 23). Ferulic acid is predominantly found in esterified form within polysaccharides, such as arabinoxylans, which are major components of cereal cell walls (24, 25). When these food components are ingested, ferulic acid is known to be released by various intestinal bacteria (26–29). Moreover, the intake of arabinoxylans has been extensively associated with health benefits in humans, primarily through the microbial production of short-chain fatty acids (SCFAs), which contribute to gut health. Ferulic acid is known to undergo reduction in the human gut and is subsequently detected as HMPA in human serum. The potential physiological effects of continuous HMPA intake have already been outlined in the Introduction.

The intake of foods containing feruloylated arabinoxylans, chlorogenic acid, and other related compounds has been suggested to result in the production of not only short-chain fatty acids (SCFAs) but also HMPA in the gut. The ferulic acid reduction pathway identified in *W. cibaria* represents a novel reductive metabolic pathway that competes with heterolactic fermentation and may contribute to intracellular redox regulation during fermentation as well as probiotic functions in the host. Plant-derived cinnamic acid derivatives appear to serve as electron acceptors during bacterial growth, supporting the anaerobic proliferation of microbes in the gut through reductive metabolism. Meanwhile, the reduced products of these cinnamic acid derivatives, such as HMPA, are increasingly being recognized through various studies for their potential roles in host health, highlighting promising avenues for future research. Together, these findings reveal a previously unrecognized redox-balancing mechanism in heterofermentative lactic acid bacteria and highlight hydroxycinnamic acids as potential electron acceptors supporting anaerobic metabolism in food- and gut-associated microbial ecosystems.

## MATERIALS AND METHODS

### Bacterial strains and growth conditions

*Weissella cibaria* JCM12495, the type strain of *W. cibaria*, was obtained from the NITE Biological Resource Center (NBRC; Kisarazu, Japan; NBRC106073) and used throughout this study. Cells were routinely cultivated at 37℃ under anaerobic conditions in screw-cap bottles containing MRS broth (Biokar Diagnostics, Beauvais, France) with gentle agitation to prevent cell sedimentation.

Ferulic acid and other hydroxycinnamic acids were dissolved in distilled water by adjusting the pH to 7.0 with KOH, followed by filter sterilization. These compounds were added to the culture medium at the indicated final concentrations (0–50 mM). Bacterial growth was monitored by measuring optical density at 610 nm.

*Escherichia coli* DH5α was used for plasmid construction, and *E. coli* BL21(DE3) was used for heterologous protein expression under the control of the T7 promoter. Recombinant *E. coli* strains were cultivated in LB medium supplemented with ampicillin (100 µg mL⁻¹).

### Analysis of ferulic acid reduction and fermentation products

Ferulic acid and the reduction product HMPA were analyzed by reversed-phase HPLC. Separation was performed using a TSK gel ODS column (TSK gel ODS-80TM, 4.6 mm i.d. × 250 mm; Tosoh Corp., Tokyo, Japan) maintained at 40°C. The mobile phase consisted of 1% (v/v) acetic acid–methanol (11:9, v/v) delivered at a flow rate of 0.8 mL min⁻¹, and the analytes were detected by UV absorbance at 280 nm. Culture samples were centrifuged at 12,000 × *g* for 5 min to remove cells, and the resulting supernatants were extracted with ethyl acetate. The organic phase was evaporated to dryness under reduced pressure, and the residue was dissolved in the HPLC mobile phase. Samples were filtered through a 0.22-µm membrane filter prior to injection.

Heterolactic fermentation products were quantified as follows. Culture supernatants were collected at the start of cultivation (0 h) and after 16 h of cultivation, centrifuged at 12,000 × *g* for 5 min, and filtered through 0.22-µm membrane filters. Lactate concentrations were determined using a Lactate Assay Kit-WST (Dojindo Laboratories, Kumamoto, Japan) according to the manufacturer’s instructions, by measuring the absorbance of the generated WST formazan at 450 nm. Ethanol concentrations were measured, after appropriate dilution with distilled water when necessary, using an EnzyChrom Ethanol Assay Kit (BioAssay Systems, Hayward, CA, USA), based on the absorbance of the produced MTT formazan at 565 nm. Absorbance measurements were performed using a microplate reader (SH-9000 Lab; Corona Electric, Hitachinaka, Japan). Acetate concentrations were determined by HPLC using connected ion-exclusion columns (TSKgel OApak-P and TSKgel OApak-A, 7.8 mm i.d. × 300 mm; Tosoh). The mobile phase was 0.75 mM sulfuric acid, delivered at a flow rate of 0.5 mL min⁻¹. The column temperature was maintained at 40°C, and detection was carried out by UV absorbance at 210 nm. Acetate was quantified using calibration curves generated with authentic standards.

### Transcriptome analysis

*Weissella cibaria* was cultivated for 6 h in MRS medium in the presence or absence of ferulic acid (10 mM). Cells were harvested by centrifugation, and total RNA was extracted using TRIzol reagent (Ambion, Thermo Fisher Scientific, Waltham, MA, USA). RNA was further purified using the Monarch Total RNA Miniprep Kit (New England Biolabs, Ipswich, MA, USA) according to the manufacturer’s instructions. RNA-seq library preparation and sequencing were performed by Genome-Lead Co., Ltd. (Takamatsu, Japan) as an outsourced service. Ribosomal RNA was removed from total RNA using the NEBNext rRNA Depletion Kit (Bacteria) (NEB). Strand-specific RNA-seq libraries were prepared using the MGI Easy RNA Directional Library Prep Set (MGI Tech Co., Ltd., Shenzhen, China). Paired-end sequencing was conducted on a DNBSEQ-G400FAST platform (MGI Tech Co., Ltd., Shenzhen, China) using the DNBSEQ-G400RS High-throughput Rapid Sequencing Set (FCS PE150; MGI Tech Co., Ltd.). Sequencing reads were mapped to the *W. cibaria* JCM12495 genome sequence (GenBank accession no. GCF_005405525.1) using Bowtie2 (version 2.4.1). RNA-seq experiments were performed in duplicate, and mean TPM values are reported (Table S1). Gene expression levels were calculated as transcripts per million (TPM), and differential expression between cultures grown in the presence and absence of ferulic acid was assessed.

### Preparation of crude extracts and enzyme assays

*Weissella cibaria* was cultivated in MRS medium containing 30 mM ferulic acid, and cells were harvested by centrifugation. The cell pellet was resuspended in 50 mM potassium phosphate buffer (pH 6.5) and disrupted by sonication using an ultrasonic disruptor (Insonator 201; Kubota Corporation, Tokyo, Japan) at 170 W for 20 min while cooling the sample with ice water. Cell debris was removed by centrifugation at 12,000 × *g* for 20 min at 4°C, and the resulting supernatant was used as a crude enzyme extract. When required, crude extracts were dialyzed overnight at 4°C against 50 mM potassium phosphate buffer (pH 6.5) using cellulose dialysis tubing. Protein concentrations were determined by the Bradford method using a Bio-Rad Protein Assay kit (Bio-Rad Laboratories, Hercules, CA, USA) with bovine serum albumin as the standard (30).

Protein concentration and enzyme removal were performed by ultrafiltration using Amicon Ultra-15 centrifugal filter units with a 10-kDa molecular weight cutoff (Ultracel-10K; Merck Millipore, Burlington, MA, USA).

Ferulate CoA-transferase activity was assayed in reaction mixtures containing enzyme solution, 50 mM potassium phosphate buffer (pH 6.5), 0.5 mM ferulic acid, and 2 mM acetyl-CoA. Reactions were incubated at 35°C, and the increase in absorbance at 345 nm was monitored using a spectrophotometer (UV-2600i; Shimadzu Corporation, Kyoto, Japan). One unit of enzyme activity was defined as the amount of enzyme that catalyzed the formation of 1 µmol of feruloyl-CoA from ferulic acid per minute. Feruloyl-CoA concentrations were calculated using the reported molar extinction coefficient (ε = 1.9 × 10⁴ L mol⁻¹ cm⁻¹) (31).

HMPA-forming activity was assayed by supplementing the reaction mixture with 2 mM FAD and 2 mM NADH. After incubation for 2 h, reactions were terminated by the addition of hydrochloric acid, and products were extracted with ethyl acetate. HMPA formation was confirmed by HPLC analysis.

### Heterologous expression, detection, and partial purification of FarA and FarB

The *farA* was amplified by PCR from the genomic DNA of *W. cibaria* using primers designed to introduce an N-terminal His-tag (Table S2) and cloned into the pRSET A expression vector (Invitrogen, Carlsbad, CA, USA). The *farB* was amplified using primers designed to introduce a C-terminal His-tag (Table S2) and cloned into the pET-21(+) vector (Novagen, Madison, WI, USA). Cloning was performed using the NEBuilder Assembly Kit (New England Biolabs), and the resulting constructs were transformed into *E. coli* DH5α. After sequence verification, the plasmids were introduced into *E. coli* BL21(DE3) for protein expression.

Expression of recombinant proteins was confirmed by SDS-polyacrylamide gel electrophoresis (SDS-PAGE) and Western blot analysis. SDS-PAGE was performed according to the method of Laemmli (32). Protein Ladder One Plus (Triple-color for SDS-PAGE; Nacalai Tesque, Kyoto, Japan) was used as a molecular weight marker. Proteins were transferred onto PVDF membranes and probed with an anti-His-tag monoclonal antibody (OGHis, mouse IgG; MBL) as the primary antibody and an HRP-conjugated goat anti-mouse IgG (H+L) antibody (Invitrogen) as the secondary antibody. Immunoreactive bands were detected using Western Blot Quant HRP Substrate (Takara Bio, Kusatsu, Japan) and visualized with an ImageQuant 500 system (Cytiva, Marlborough, MA, USA).

Recombinant FarA and FarB proteins were partially purified by Ni-affinity chromatography using a HisTrap HP 5 mL column connected to an ÄKTA start chromatography system (Cytiva).

### LC-QTOF-MS analysis

Samples (1 mL) passed through a pre-conditioned Sep-Pak tC18 Plus Short cartridge (400 mg; Waters, Milford, MA, USA) and washed with 4 mL of water-methanol (50:50, v/v). The combined flow-through was diluted with water to 10 mL and used directly for CoA analysis or further diluted 10-fold for ferulic acid and HMPA analysis. All samples were filtered through a 0.2-µm PTFE membrane prior to LC-QTOF-MS analysis.

Chromatography was performed using an ACQUITY UPLC H-Class PLUS system coupled to a Xevo G3 QTof mass spectrometer (Waters). Analytes were separated on an ACQUITY Premier BEH C18 column (2.1 mm i.d. × 150 mm, 1.7 µm; Waters) maintained at 30°C. The mobile phases consisted of (A) 20 mM ammonium acetate (pH 6.8) and (B) acetonitrile, delivered at a flow rate of 0.30 mL min^−1^ using a linear gradient. The injection volume was 0.2 µL. Mass spectrometry was performed using electrospray ionization (ESI). CoA was analyzed in positive-ion mode, while ferulic acid and HMPA were analyzed in negative-ion mode. Data were acquired over an *m*/*z* range of 50–2,000.

## SUPPLEMENTAL MATERIAL

Supplemental material aem.00111-26-s0001.pdf Fig. S1 to S3; Tables S1 and S2

ASM does not own the copyrights to Supplemental Material that may be linked to, or accessed through, an article. The authors have granted ASM a non-exclusive, world-wide license to publish the Supplemental Material files. Please contact the corresponding author directly for reuse.
