# Supplementary material for: Reduction of ferulic acid as an electron acceptor under anaerobic conditions by the heterofermentative lactic acid bacterium Weissella cibaria
Source: Appl Environ Microbiol. 2026 Mar 30;92(4):e00111-26. doi: 10.1128/aem.00111-26 (PMC13101492; doi:10.1128/aem.00111-26)
Supplement: Supplemental material — Fig. S1 to S3; Tables S1 and S2 [file aem.00111-26-s0001.pdf]

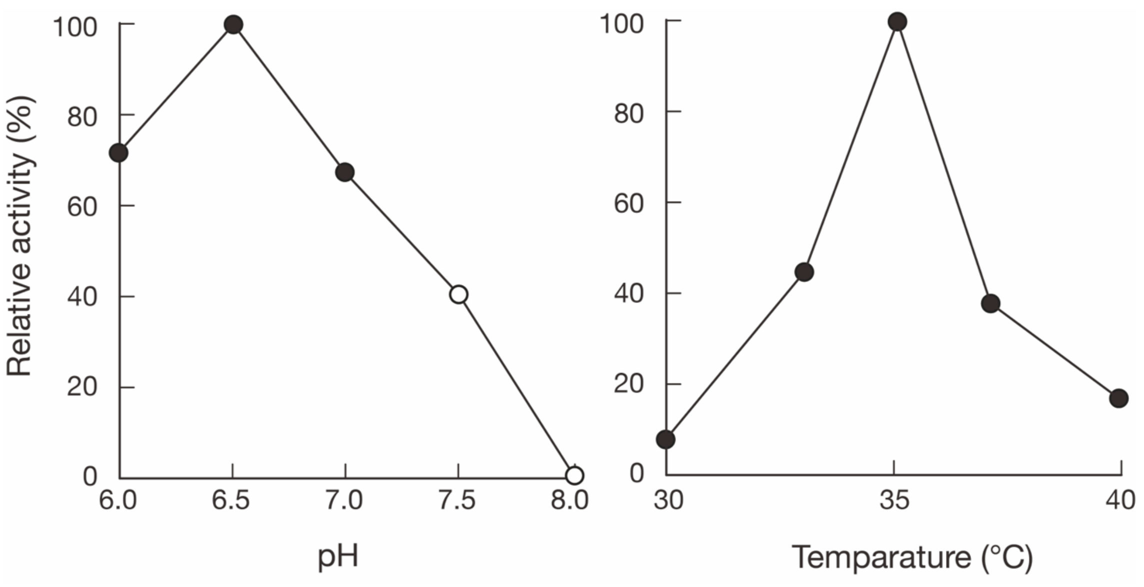

Fig. S1. Effects of pH and temperature on FarA activity. To determine appropriate conditions for enzymatic assays, FarA activity was examined in preliminary experiments. Representative data from a single experiment are shown. (Left) pH dependence of FarA activity measured using 50 mM potassium phosphate buffer (●) and 50 mM Tris-HCl buffer (○). (Right) Temperature dependence of FarA activity measured in 50 mM potassium phosphate buffer (pH 6.5).

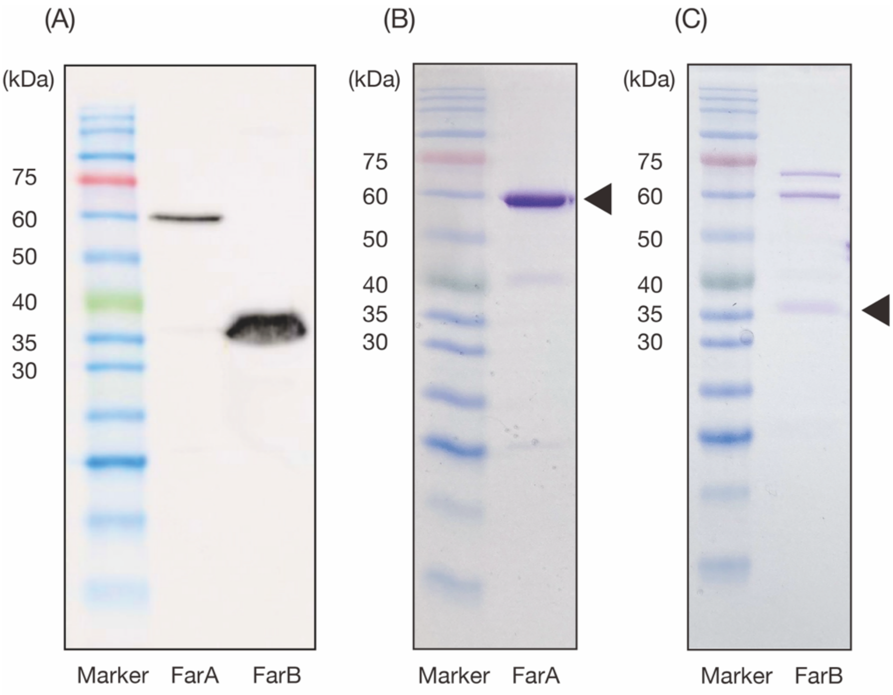

Fig. S2. Expression and purification of recombinant FarA and FarB proteins.

(A) Western blot analysis of His-tagged FarA and FarB expressed in *Escherichia coli*. Proteins were detected using an anti-His-tag monoclonal antibody. Immunoreactive bands corresponding to FarA and FarB were observed at approximately 60 kDa and 37 kDa, respectively. (B, C) SDS–PAGE analysis of partially purified FarA (B) and FarB (C) after Ni-affinity chromatography. The bands corresponding to FarA and FarB are indicated by arrowheads and match the bands detected by Western blotting. Molecular mass markers are indicated on the left (kDa).

(A)

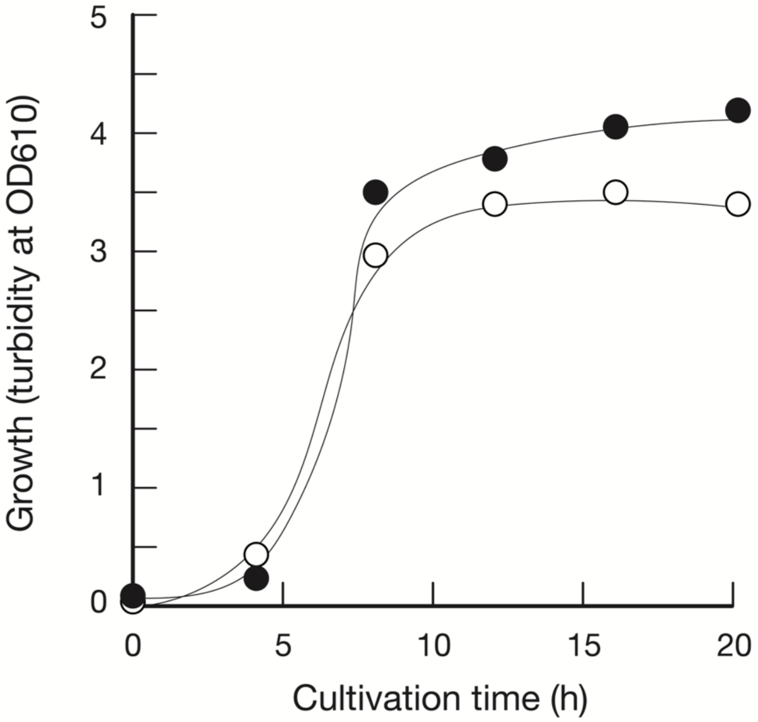

(B)

Ferulic acid (mM)	OD610		
	0 h	8 h	16 h
0	0.04	2.98	3.51
10	0.02	3.47	4.06
50	0.04	—	3.70

Fig. S3. Effect of ferulic acid on the growth of *Weissella cibaria*. (A) Growth curves of *W. cibaria* cultured in the presence of 0 mM (open circles) or 10 mM (closed circles) ferulic acid. (B) Optical density measurements of cultures grown with different concentrations of ferulic acid. Growth at 50 mM ferulic acid was evaluated only at the endpoint after 16 h of cultivation.

Table S1. Transcriptional induction of *farA*, *farB* and *farR* by ferulic acid in *Weissella cibaria* JCM12495

Locus tag	Gene	TPM (mean)		Ratio
		+Ferulic acid	–Ferulic acid	
FFM76_RS04205	*farA*	6288.2	107.3	58.1
FFM76_RS04210	*farB*	9630.6	189.0	50.7
FFM76_RS04200	*farR*	335.7	157.0	2.1

a TPM values represent the mean of two independent RNA-seq experiments (n = 2) and are shown rounded to one decimal place. Ratios were calculated using unrounded TPM values.

Table S2. Primers used for expression of FarA and FarB.

Primer name	Target gene	Sequence (5’-3’)
farA_fw	*farA*	TGACGATAAGGATCGATGGGGTATGAGCAAGG TTATTTCAGCAA
farA_rv	*farA*	TCGACGTCTAGAGCTCGAGCAATTTAAAGACTA ACCAATTGTTCC
farB_fw	*farB*	CGACGGAGCTCGAATTCGAATACGTAATCTTCA AATTCTTAAC
farB_rv	*farB*	CGCCTATTGTTAAGGGGAGATCATTGCTCTATT TTTCCTCTGT

a The primers for *farA* and *farB* contain sequences for assembly into the BamHI sites of pRSET A and pET21(+), respectively.
